# Antenatal screening for Down’s syndrome: Revised nuchal translucency upper truncation limit due to improved precision of measurement

**DOI:** 10.1177/0969141320937321

**Published:** 2020-07-01

**Authors:** Stephen H Vale, Wayne J Huttly, Nicholas J Wald

**Affiliations:** 1 Logical Medical Systems Ltd, London, UK; 2 Wolfson Institute of Preventive Medicine, Barts and the London School of Medicine and Dentistry, Queen Mary University of London, London, UK; 3 UCL Institute of Health Informatics, London, UK

**Keywords:** Down’s syndrome, antenatal screening, nuchal translucency, truncation limit, prenatal screening, validation plot

## Abstract

**Objective:**

To determine whether the improved precision of nuchal translucency (NT) measurement used in antenatal screening for Down’s syndrome observed over time as evidenced by a decrease in the multiple of the median (MoM) standard deviation requires a modification to the NT MoM truncation limits to maintain accurate risk estimation.

**Methods:**

Probability plots were derived from the measurements of NT MoM values used in a 2018 audit of 22,362 unaffected pregnancies. The plots were used to determine whether the NT MoM upper truncation limit should be lowered. Validation plots were used to assess the screening accuracy of Down’s syndrome risk estimates calculated from observed NT MoM values in the 22,362 unaffected pregnancies and 69 Down’s syndrome pregnancies for original and revised NT MoM truncation limits.

**Results:**

Probability plots indicated that with improved precision of NT measurements, there was deviation from a Gaussian distribution at less high MoM values than with less precise measurements. Validation plots showed that using the current NT MoM upper truncation limit of 2.5 MoM with improved precision NT measurements overestimates the Down’s syndrome risk (median risk in highest risk category expressed as an odds was 53.3:1 and observed prevalence was 1:1.1). The large discrepancy was corrected by changing the NT upper truncation limit to 2.0 MoM (median risk in highest risk category expressed as an odds was 1:1.78 and observed prevalence 1:2.7).

**Conclusion:**

The NT MoM upper truncation limit should be reduced from 2.5 to 2.0 MoM.

## Introduction

Measurement of nuchal translucency (NT) is an important marker in antenatal screening for Down’s syndrome, trisomy 18 and trisomy 13. Over time, the precision of measuring NT has improved leading to a reduction in the standard deviation. For example, at 12 completed weeks’ gestation, the log_10_ NT MoM (multiple of the median) standard deviation was 0.1329, 0.1105 and 0.0855 in 2003,^1^ 2010^2^ and 2018,^3,4^ respectively, as reported from the Wolfson Institute Screening Service. The distribution of NT is positively skewed; after log transformation, the distribution is reasonably Gaussian, but there is still a degree of positive skew.^5^ To deal with screening markers deviating from Gaussian distributions in the tails of the distributions, it is standard practice to specify truncation limits in which values above or below these limits are assigned values at the truncation limits.^6,7^

As NT measurement has become more precise, there is a case for lowering the upper truncation limit from the 2.5 MoM value^2^ set in 2010 to take account of the positively skewed distribution of log-transformed NT MoM values. We here examined data from the Wolfson Institute of Preventive Medicine Antenatal Screening Programme to determine whether there was a need to revise the NT upper truncation limit.

## Methods

Data from a 2018 audit of antenatal screening were used based on measurements from 22,362 unaffected singleton pregnancies and 69 Down’s syndrome pregnancies.^8^ A probability plot was generated for the NT MoM values and inspected to determine if the upper truncation limits should be lowered from the currently specified value of 2.50 MoM.^2^ A term Down’s syndrome risk estimate was calculated from the NT measurements for each of the 22,362 unaffected singleton pregnancies and 69 affected singleton pregnancies. The risk estimates were calculated by previously described standard methods^9^ from the overlapping log_10_ NT MoM Gaussian distributions of affected and unaffected pregnancies, using published sets of NT distribution parameters^2,4,10^ (see Appendix 1), the observed overall Down’s syndrome prevalence in the sample population and the currently specified NT upper truncation limits of 2.50 MoM and 2.0 MoM.

To assess the accuracy of risk estimation using the two NT upper truncation limits, validation plots^11,12^ were created in which the risk estimates were ranked and put into seven categories with approximately equal numbers of affected pregnancies in each category. Within each category, the median risk and the observed prevalence of Down’s syndrome were calculated. The accuracy of risk estimation is displayed by plotting the median risk against the observed prevalence in each category. Points lying close to the diagonal line of identity (where predicted risk equals observed prevalence) indicate the accuracy of risk estimation.

Statistical analyses were performed using RStudio (RStudio Team (2019). RStudio: Integrated Development for R. RStudio, Inc., Boston, MA URL http://www.rstudio.com/). NT MoM values calculated at the time of screening with the Alpha software (Logical Medical Systems, London, UK) were used.

## Results

**Figure 1.**
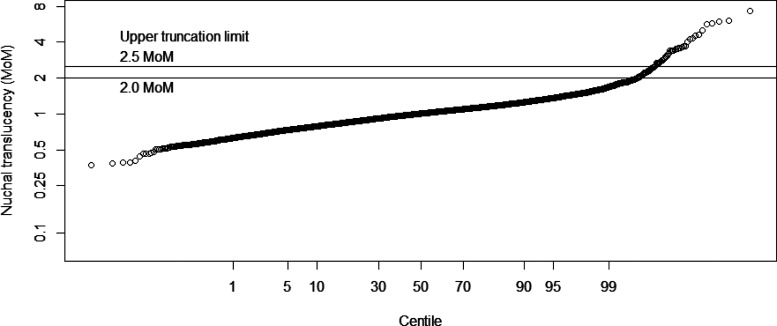
Probability plot of NT measurements in unaffected pregnancies from 2018 study.^8^ The two horizontal lines show the positions of the NT upper truncation limits at 2.0 and 2.5 MoM. MoM: multiple of the median.

Figure 1 shows the NT MoM probability plot. Deviation from a log-Gaussian distribution is apparent at an NT measurement of about 2.0 MoM rather than 2.5 MoM.

**Figure 2.**
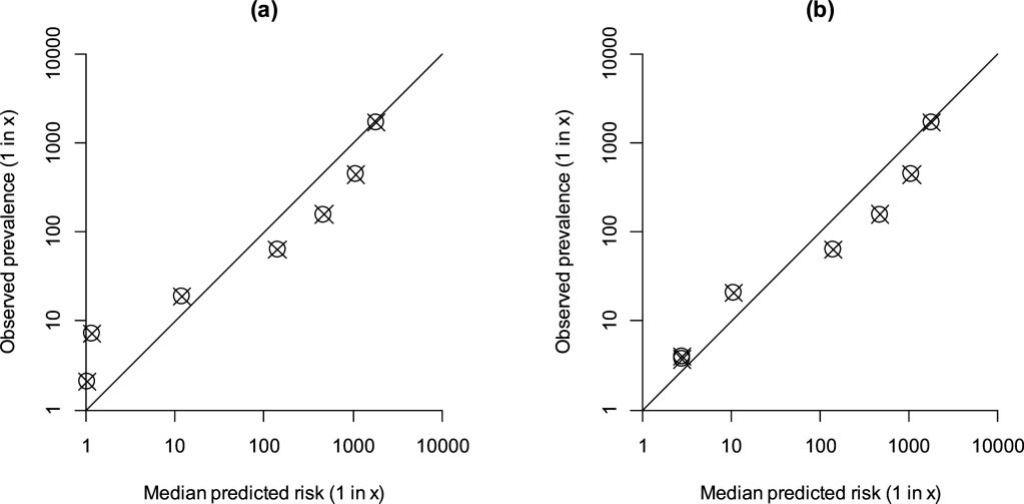
Validation plots for Down’s syndrome risk estimates expressed as probabilities (1 in x) calculated using NT measurements from the 2018 audit,^8^ with the current reduced unaffected NT standard deviation.^4^ (a) Validation plot with NT upper truncation limit of 2.5 MoM. (b) Validation plot with NT upper truncation limit of 2.0 MoM. (The points plotted in the two highest risk categories are nearly superimposed.).

Figure 2(a) and (b) shows validation plots for Down’s syndrome risk estimates calculated using the current NT upper truncation limits of 2.5 MoM and 2.0 MoM, respectively. Table 1 gives the numerical results shown in Figure 2(a) and (b). When the NT upper truncation limit is 2.5 MoM, the risk estimates are inaccurate at high risks (greater than about 1 in 10). In the highest risk category, over 50 times more affected pregnancies are predicted than observed: the predicted risk estimate and the observed prevalence, expressed as an odds (affected:unaffected) are 53.1:1 and 1:1.1, respectively.

**Table 1.** Numerical results for the validation plot in Figure 2.

Predicted risk			Observed			
	Median				Prevalence	
Category	Probability	Odds	Down’s Syndrome	Unaffected	Proportion	Odds
(a) NT upper truncation limit = 2.5 MoM						
>1 in1.0195	1 in 1.0188	53.3:1	10	11	1 in 2.1	1:1.1
1 in 1.0195–	1 in 1.166	6.0:1	10	62	1 in 7.2	1:6.2
1 in 1.26–	1 in 12.0	1:11.0	10	180	1 in 19.0	1:18.0
1 in 35.4–	1 in 141.2	1:140.2	10	630	1 in 64.0	1:63.0
1 in 245–	1 in 472	1:471	10	1571	1 in 158	1:157
1 in 658–	1 in 1077	1:1076	10	4439	1 in 445	1:444
<1 in1352	1 in 1816	1:1815	9	15469	1 in 1720	1:1719
All	1 in 1661	1:1660	69	22362	1 in 325	1:324
(b) NT upper truncation limit = 2.0 MoM						
>1 in 2.7864	1 in 2.7831	1:1.7831	10	27	1 in 3.7	1:2.7
1 in 2.7864–	1 in 2.796	1:1.796	10	30	1 in 4.0	1:3.0
1 in 2.91–	1 in 10.7	1:9.7	10	196	1 in 20.6	1:19.6
1 in 35.4–	1 in 141.2	1:140.2	10	630	1 in 64.0	1:63.0
1 in 245–	1 in 472	1:471	10	1571	1 in 158	1:157
1 in 658–	1 in 1077	1:1076	10	4439	1 in 445	1:444
<1 in 1352	1 in 1816	1:1815	9	15,469	1 in 1720	1:1719
All	1 in 1661	1:1660	69	22,362	1 in 325	1:324

NT: nuchal translucency; MoM: multiple of the median.

Reducing the NT upper truncation limit to 2.0 MoM brings the plotted points closer to the diagonal line of identity and thereby improves the accuracy of the risk estimates. With an upper truncation limit of 2.0 MoM in the highest risk category, the predicted risk estimate and the observed prevalence (both expressed as an odds) are 1:1.78 and 1:2.7, respectively.

## Discussion

This study shows that the NT MoM upper truncation limit should be revised from 2.5 to 2.0 MoM as a result of the improved prevision of NT measurements over time. The less high upper truncation limit improves the accuracy of risk estimation in antenatal screening for Down’s syndrome. Retaining the 2.5 MoM upper truncation limit overestimates risk estimation for pregnancies with high NT MoM values. Although such pregnancies would probably be screen-positive, a truncation limit of 2.0 MoM avoids an excess of very high inaccurate risk estimates among women with screen-positive results.

The distribution of NT log_10_ MoMs is positively skewed, so that the distribution of NT measurements at higher MoM values (those above about 2.0 MoM) is not well estimated by a Gaussian distribution (applicable to about 0.4% of unaffected pregnancies). With a smaller standard deviation, the expected number of pregnancies with high NT MoMs is substantially less than that observed. Consequently, the likelihood ratio calculated from the overlapping Gaussian distributions of affected and unaffected pregnancies at high NT MoMs will be overestimated, and hence the risk of having an affected pregnancy will be overestimated. This is illustrated in the validation plot of Figure 2(a) where the NT MoM upper truncation limit is set at 2.5 MoM, which shows how the error is limited to women with the highest risk estimates.

**Figure 3.**
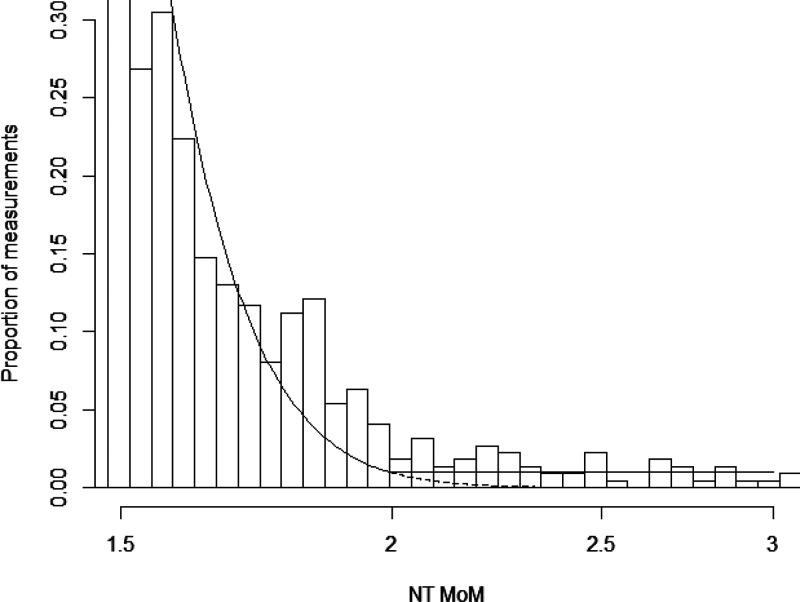
Partial histogram of NT MoM values (between 1.5 and 3.0 MoM) in unaffected pregnancies from the 2018 audit.^8^ The solid dotted line shows the Gaussian distribution of NT measurements. To show a single line, a weighted average for gestational age weeks 10 to 13 was used to calculate the NT MoM standard deviation. The horizontal line is specified by the truncation limit (2.0 MoM). MoM: multiple of the median.

Figure 3 shows a partial histogram of NT MoM values (between 1.5 and 3.0 MoM) in unaffected pregnancies. The figure also shows the proportions using a Gaussian distribution with and without truncation at 2.0 MoM. With an NT upper truncation limit of 2.0 MoM, the truncated NT distribution fits the observed data reasonably well, illustrating how it leads to improved accuracy of risk estimation.

Revising the NT upper truncation limit from 2.5 MoM to 2.0 MoM will not reduce the detection rate because the effect is limited to pregnancies with very high risk estimates (greater than about 1 in 10) that would anyway be classified as screen positive. It, however, avoids the overestimation of risk among some pregnancies with the highest risk estimates and being limited in this way is robust to centre-to-centre variation in NT precision.

The improvement in the accuracy of risk estimation using a less high NT upper truncation limit of 2.0 MoM will apply to screening for trisomy 18 and trisomy 13 as well as for Down’s syndrome because the over estimation of risk for high NT MoM values arises from the persistence of right skewness in the distribution of log_10_ NT MoM values in unaffected pregnancies.

In conclusion, the NT MoM upper truncation limit should be revised from 2.5 to 2.0 MoM when used with the smaller NT standard deviation associated with the improved precision of current NT measurements.

## Appendix 1. Summary of published NT distribution parameters.^2,4,10^

**Table:**

	Down’s syndrome pregnancies	Unaffected
Median (log_10_ MoM)	0.8554679–0.0064686 × gestational age in days	0.0000
**Standard deviation (log_10_ MoM)**		
10 completed weeks	0.2313	0.1193^a^
11 completed weeks		0.0976
12 completed weeks		0.0855
13 completed weeks		0.0855
**Lower truncation limits (MoM)**		
10 completed weeks	0.5	
11 completed weeks	0.7	
12 completed weeks	0.8	
13 completed weeks	0.85	

MoM: multiple of the median.

^a^Estimated from the 10-week standard deviation in Bestwick et al.^2^ decreased by the reduction in standard deviation at 11 and 12 weeks in Wald et al.^4^ compared to Bestwick et al.^2^
